# Cavernous Hemangioma of the Small Bowel: A Case Report and Literature Review

**DOI:** 10.7759/cureus.3113

**Published:** 2018-08-06

**Authors:** Ceren Durer, Seren Durer, Mohamad Sharbatji, Isin Y Comba, Ilan Aharoni, Umair Majeed

**Affiliations:** 1 Internal Medicine, Florida Hospital-Orlando, Orlando, USA; 2 Internal Medicine, UCF Internal Medicine Residency Program, Orlando, USA; 3 Gastroenterology, Florida Hospital, Orlando, USA; 4 Internal Medicine Residency, Florida Hospital-Orlando, Casselberry, USA

**Keywords:** cavernous hemangioma, capsule endoscopy, double balloon enteroscopy, chronic anemia

## Abstract

Hemangiomas of the small intestine are rare and very difficult to diagnose preoperatively. Clinical presentations may include occult or massive gastrointestinal (GI) bleeding, obstruction, intussusception, and perforation. We report a 66-year-old Caucasian male patient with severe anemia secondary to occult GI bleeding from a cavernous hemangioma in the jejunum. A double balloon enteroscopy following capsule endoscopy was performed to obtain biopsy samples, which established the final diagnosis.

## Introduction

Hemangioma of the small bowel is an uncommon condition, accounting for 5 to 10% of all benign lesions of the small bowel [1]. It usually presents in young people with no sex predilection. Intestinal hemangiomas may cause occult or massive gastrointestinal bleeding, obstruction, intussusception, and small bowel perforation [2]. According to the size of affected vessels, hemangiomas are histologically classified into cavernous, capillary or mixed-type, with the cavernous type being the most common. In the gastrointestinal tract, the most commonly involved site is the jejunum. We report a case of cavernous hemangioma of the jejunum presenting with severe anemia. No remarkable findings were detected in upper and lower endoscopies. Correspondingly, the patient underwent wireless capsule endoscopy and double-balloon enteroscopy, which demonstrated a 25-mm submucosal mass with mild superficial ulceration/erosions of the surface mucosa. The pathology demonstrated that the lesion was consistent with cavernous hemangioma without evidence of malignancy.

## Case presentation

**Figure 1:**
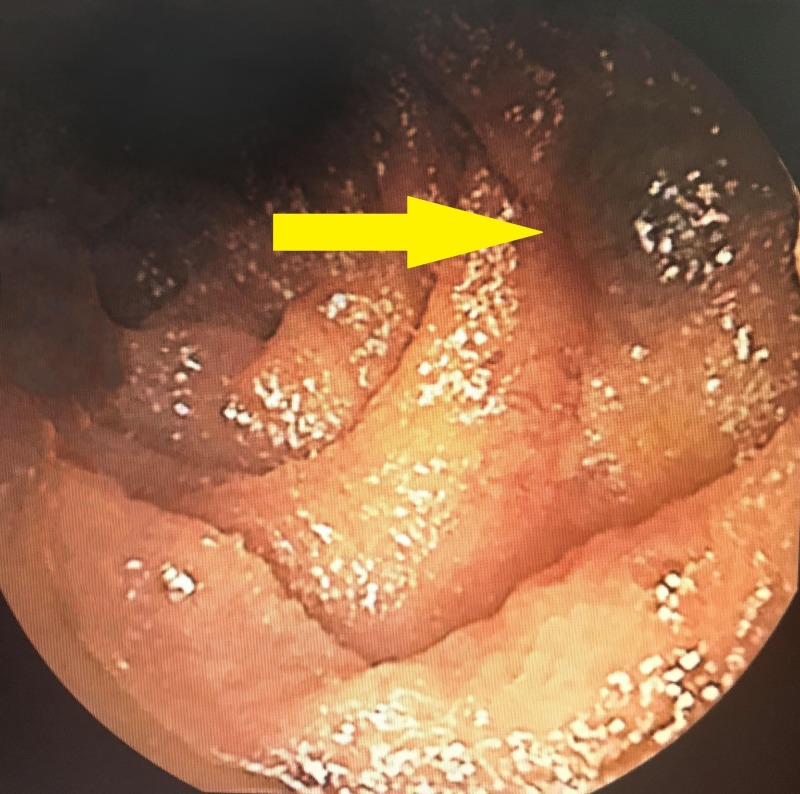
Capsule endoscopy

A 66-year-old male presented to the emergency room due to worsening leg pain. His past medical history was significant for peripheral artery disease and iron deficiency anemia. Initial laboratory tests revealed an unexpectedly low hemoglobin level of 5.4 g/dl. He received three units of packed red blood cells and subsequently the hemoglobin level increased to 6.9 g/dl. Our gastroenterology department was consulted for evaluation of occult gastrointestinal bleeding. There was no hematochezia, melena, hematemesis, fatigue, or abdominal pain. The patient had been taking oral iron supplementation for the last five years for iron deficiency anemia. Previous upper and lower endoscopies were negative. On physical examination, the patient had pale conjunctivae. The abdomen was noted to be soft and non-tender. No masses, organomegaly or vascular bruits were detectable. The vital signs were stable, and the laboratory investigations were as follows: a hemoglobin (hb) level of 6.9 g/dL, a mean corpuscular volume of 73.5, a hematocrit level of 22.7% with normal white blood cell and platelet counts. The analyses for iron-deficiency anemia showed ferritin levels of 6 ng/mL, serum iron levels of 25 μg/dL, total iron-binding capacity of 535 μg/dL, and transferrin saturation of 5%. Upper and lower endoscopy showed no active bleeding or suspicious lesions. A small bowel capsule endoscopy was performed, which revealed a suspicious lesion over the jejunum with evidence of fresh blood (Figure 1).

For further investigation, double balloon enteroscopy was performed, which revealed a proximal jejunal soft submucosal mass (25 mm) with mild superficial ulceration/erosions of the surface mucosa. Biopsy samples were taken from the mass and the patient was referred for further management and surgical evaluation. The final pathology results revealed a cavernous hemangioma without evidence of malignancy.

## Discussion

Cavernous hemangioma is one of the congenital benign vascular lesions and is not a true tumor. It can be solitary, multiple, or associated with various syndromes such as blue rubber bleb nevus syndrome, Klippel-Trenaunay-Weber syndrome, and Maffucci syndrome [3]. Cavernous hemangioma is a soft, compressible, bluish purple lesion and consists of blood-filled spaces (cavern) between dilated vessels within the mucosa and submucosa. Its size may range from a few millimeters to several centimeters. Hemangioma, telangiectasia, angiodysplasia, and phlebectasia are typical forms of small bowel vascular lesions.

Preoperative diagnosis is extremely difficult because small hemangiomas are rarely demonstrable with traditional techniques such as upper and lower endoscopies. However, several imaging modalities including wireless capsule endoscopy, double balloon enteroscopy, multiphase computed tomography enterography (CTE), magnetic resonance enterography (MRE) are currently available options to investigate small bowel lesions [4]. Small bowel capsule endoscopy is a noninvasive imaging test and can be recommended when the source of bleeding remains unidentified after upper and lower endoscopy. On the other hand, double balloon enteroscopy is an invasive and highly sensitive diagnostic tool providing both therapeutic and diagnostic interventions. In the current case, the patient had severe chronic anemia without evidence of gross GI bleeding. Small bowel capsule endoscopy demonstrated suspicious findings in the jejunum and double-balloon enteroscopy helped diagnose the bluish submucosal mass in the small bowel.

A comprehensive search of electronic databases (PubMed, Embase, and the Web of Science databases) was performed to identify studies published from January 1, 2000 until June 30, 2018 using MeSH terms and keywords such as ‘cavernous hemangioma’, ‘wireless capsule endoscopy’, ‘double balloon enteroscopy’, ‘anemia’ and ‘lower gastrointestinal bleeding’. Reference lists were screened to identify additional relevant studies. A standardized form was used for data extraction. There have been about 15 cases of uncomplicated cavernous hemangioma of small intestine diagnosed preoperatively [1, 5-18]. Patient information is summarized in Table 1. Most patients were male with age ranging from 9 to 74 years. The jejunum was the most common site of small intestinal hemangiomas, and the initial clinical presentation was anemia in the majority of the cases. The diameter of the lesions varied from 0.8 cm to 50 cm. Most studies showed suspicious findings on capsule endoscopy and the lesions were resected surgically or endoscopically [1, 5-7, 9-14, 16-18]. Ejtehadi et al. demonstrated a hemangioma on scintigraphy and CT enterography while no positive finding was found on capsule endoscopy [15]; another reported case was diagnosed with contrast-enhanced CT and MRI preoperatively [8]. Cavernous hemangioma can also present with other clinical symptoms and complications. Abdul Aziz et al. reported a six-year-old patient with abdominal pain and distention who developed subacute intestinal obstruction secondary to cavernous hemangioma (15 cm) after blunt trauma to the abdomen [2]. Open surgical excision of the hemangioma along with affected small bowel was performed. In a guideline by the American Gastroenterological Association (AGA) in 2007 about the algorithms for the diagnosis and management of obscure gastrointestinal bleeding, capsule endoscopy is considered as the first-line procedure for the initial examination of small bowel after normal upper and lower endoscopies [19]. When positive findings are obtained on capsule endoscopy, double balloon enteroscopy or push enteroscopy is recommended for further management when there is no evidence of obstruction [19]. The American College of Gastroenterology (ACG) clinical guideline in 2015 also recommends the use of capsule endoscopy followed by deep enteroscopy based on findings on capsule endoscopy [20]. In the presented case, capsule endoscopy and balloon enteroscopy were performed according to these guidelines.

**Table 1:** Reports on small bowel cavernous hemangioma diagnosed preoperatively between 2000 and 2018

Author, Year	Age (Y)	Sex	Presentation	Preoperative diagnosis study	Hemangioma size (cm)	Hemangioma location	Treatment
Kazama et al. 2000 [8]	64	M	Anemia	Enteroclysis, contrast CT MRI	3.0 and 0.5	Jejunum	Surgery
Magnano et al. 2005 [1]	13	M	Fatigue, weakness anemia	Capsule endoscopy	2.0	Ileum	Laparotomy
Quentin et al. 2007 [9]	32	F	Hematochezia	Capsule endoscopy	2.0	Ileum	Laparotomy
Willert et al. 2008 [10]	19	M	Anemia	Capsule endoscopy+ balloon enteroscopy	0.8, 0.8 and 1.4	Jejunum, ileocecal valve	Endoscopic treatment
Pinho et al. 2008 [5]	9	F	Fatigue, dizziness, Anemia, melena	Capsule endoscopy	2.5	Ileum	Segmental resection
Chen et al. 2009 [7]	23	M	Anemia	Capsule endoscopy	3.0	Ileum	Laparoscopic treatment
Elias et al. 2010 [14]	39	M	Anemia	Capsule endoscopy+ push enteroscopy	NA	Jejunum	Laparotomy
Huber et al. 2012 [6]	23	M	Melena, anemia	Capsule endoscopy+ balloon enteroscopy	3.0	Jejunum	Laparoscopic operation
Pera et al. 2012 [18]	16	M	Anemia, palpitation, fatigue	Capsule endoscopy+ balloon enteroscopy	4.3	Jejunum	Laparoscopic operation
Ersoy et al. 2013 [13]	50	F	Hematemesis, melena	Capsule endoscopy+ balloon enteroscopy	NA	Jejunum	Segmental resection
Fernandes et al. 2014 [12]	56	F	hematochezia syncope, anemia	Capsule endoscopy+ CT enterography	14	Ileum	Laparotomy
Bae et al. 2015 [16]	13	M	Nausea, dizziness, anemia	Capsule endoscopy	5.2	Jejunum	Excision of the mass*
Peng et al. 2016 [11]	47	M	Weakness, melena	Capsule endoscopy+ Contrast-enhanced CT	50	Ileum	Laparotomy
Akazawa et al. 2016 [17]	56	F	Melena	Capsule endoscopy+ balloon enteroscopy	1.3	Jejunum	Laparoscopic operation
Ejtehadi et al. 2017 [15]	40	M	Fatigue, palpitation	Scintigraphy+ CT enterography	2.6	Jejunum	Laparoscopic operation

## Conclusions

Cavernous hemangioma of the small bowel is a benign vascular lesion that can cause chronic GI bleeding presenting as chronic severe anemia. Clinical suspicion of bleeding originating from the small bowel is essential in the diagnosis of these lesions as they warrant a comprehensive workup. Based on the literature and guideline review, we recommend that capsule endoscopy followed by therapeutic double balloon enteroscopy be considered in those patients.
